# *catena*-Poly[[(nitrato-κ^2^ *O*,*O*′)silver(I)]-μ-*N*,*N*′-bis­(3-pyridyl­methyl­idene)benzene-1,4-diamine]

**DOI:** 10.1107/S1600536810025997

**Published:** 2010-07-07

**Authors:** Yong-Hao Liu, Quan Xu, Zhi-You Han

**Affiliations:** a Department of Physics, Daqing Normal University, Daqing, 163712, People’s Republic of China

## Abstract

In the title compound, [Ag(NO_3_)(C_18_H_14_N_4_)]_*n*_, the Ag^I^ atom is coordinated by two N atoms from two *N*,*N*′-bis­(3-pyridyl­methyl­idene)benzene-1,4-diamine (bpbd) mol­ecules and two O atoms from a bidentate nitrate anion. The bpbd mol­ecules bridge the Ag atoms into a chain. Two adjacent chains are further connected by Ag⋯Ag inter­actions [3.1631 (8) Å], forming a double-chain structure. A π–π inter­action [centroid–centroid distance = 3.758 (3) Å] occurs between the double chains. Inter­chain C—H⋯O hydrogen bonds are observed.

## Related literature

For general background to metal–organic frameworks with bipyridine-type ligands, see: Biradha *et al.* (2006 ▶); Cunha-Silva *et al.* (2006 ▶); Lu *et al.* (2002 ▶); Ye *et al.* (2004 ▶).
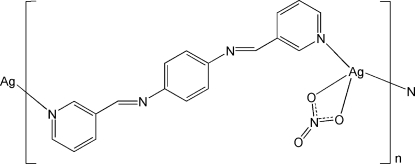


## Experimental

### 

#### Crystal data

[Ag(NO_3_)(C_18_H_14_N_4_)]
*M* *_r_* = 456.21
Triclinic, 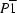

*a* = 9.2148 (18) Å
*b* = 9.771 (2) Å
*c* = 10.800 (2) Å
α = 81.51 (3)°
β = 74.27 (3)°
γ = 66.52 (3)°
*V* = 857.6 (4) Å^3^
*Z* = 2
Mo *K*α radiation
μ = 1.21 mm^−1^
*T* = 293 K
0.31 × 0.30 × 0.08 mm

#### Data collection

Rigaku R-AXIS RAPID diffractometer
Absorption correction: multi-scan (*ABSCOR*; Higashi, 1995 ▶) *T*_min_ = 0.705, *T*_max_ = 0.908
8493 measured reflections
3891 independent reflections
3163 reflections with *I* > 2σ(*I*)
*R*_int_ = 0.019

#### Refinement

*R*[*F* ^2^ > 2σ(*F* ^2^)] = 0.032
*wR*(*F* ^2^) = 0.094
*S* = 1.10
3891 reflections
244 parameters
H-atom parameters constrained
Δρ_max_ = 0.72 e Å^−3^
Δρ_min_ = −0.29 e Å^−3^

### 

Data collection: *RAPID-AUTO* (Rigaku, 1998 ▶); cell refinement: *RAPID-AUTO*; data reduction: *RAPID-AUTO*; program(s) used to solve structure: *SHELXS97* (Sheldrick, 2008 ▶); program(s) used to refine structure: *SHELXL97* (Sheldrick, 2008 ▶); molecular graphics: *DIAMOND* (Brandenburg, 1999 ▶); software used to prepare material for publication: *SHELXL97*.

## Supplementary Material

Crystal structure: contains datablocks I, global. DOI: 10.1107/S1600536810025997/hy2324sup1.cif

Structure factors: contains datablocks I. DOI: 10.1107/S1600536810025997/hy2324Isup2.hkl

Additional supplementary materials:  crystallographic information; 3D view; checkCIF report

## Figures and Tables

**Table 1:** Selected bond lengths (Å)

Ag1—N1	2.162 (2)
Ag1—N4^i^	2.163 (2)
Ag1—O2	2.731 (3)
Ag1—O3	2.704 (3)

Symmetry code: (i) 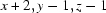.

**Table 2:** Hydrogen-bond geometry (Å, °)

*D*—H⋯*A*	*D*—H	H⋯*A*	*D*⋯*A*	*D*—H⋯*A*
C1—H1*A*⋯O3^ii^	0.93	2.50	3.276 (4)	141
C16—H16*A*⋯O2^iii^	0.93	2.44	3.280 (4)	151

Symmetry codes: (ii) 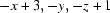; (iii) 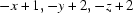.

**Fig. 1.**
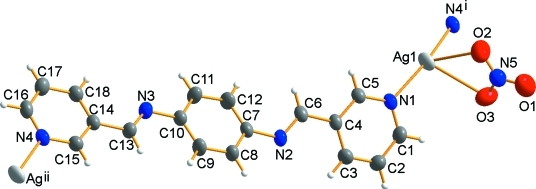
The asymmetric unit of the title compound with displacement ellipsoids shown at the 50% probability level. [Symmetry codes: (i) 2+x, -1+y, -1+z; (ii) -2+x, 1+y, 1+z.]

**Fig. 2.**
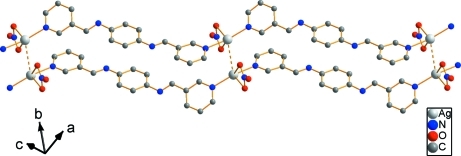
A view of the double-chain structure in the title compound.

## supplementary crystallographic information

### Comment

Bipyridine-type ligands have been extensively investigated in recent years, owing to their simple structures, readily availabilities and more predictable formation of network structures (Biradha *et al.*, 2006; Cunha-Silva *et al.*, 2006; Lu *et al.*, 2002). Moreover, when introducing double Schiff-base, a great deal of metal–organic frameworks with unusual network patterns and novel properties can be achieved due to the specific geometry including the different relative orientation of N-donors and the zigzag conformation of the space moiety between the two terminal coordination groups (Ye *et al.*, 2004).

Herein, we choose 3,3'-bipyridine-type Schiff-base as an organic linking ligand. In this simple compound, N═C—H group can act as a hydrogen bonding donor and the pyridyl N atom as an acceptor. In the title complex, the ligand takes a bidentate bridging coordination fashion and links two Ag^I^ centers with two pyridyl N atoms (Fig. 1), forming a zigzag chain, with Ag—N distances being 2.162 (2) and 2.163 (2) Å (Table 1). The two neighboring Ag atoms exhibit an Ag···Ag interaction, with a distance of 3.1631 (8) Å. The Ag atoms are also coordinated by distant nitrate O atoms [Ag—O = 2.731 (3) and 2.704 (3) Å], leading to a deviation from linearity [N—Ag—N = 159.44 (9)°]. Two adjacent chains are connected by the Ag···Ag interactions into a double-chain structure (Fig. 2). A π–π interaction [centroid–centroid distance = 3.758 (3) Å] occurs between the double-chains. Interchain C—H···O hydrogen bonds are observed (Table 2).

### Experimental

The ligand *L* was prepared according to the previous method (Ye *et al.*,  2004). 1,4-Diaminobenzene (2.14 mg, 10 mmol) was dissolved in methanol (20 ml), followed by addition of 3-pyridinecarboxaldehyde (4.24 mg, 40 mmol). The mixture was stirred at room temperature for 2 h and filtered. The resulting yellow crystalline solid was washed with methanol several times and dried in air. A solution of AgNO_3_ (33.9 mg, 0.2 mmol) in acetonitrile (10 ml) was slowly layered onto a solution of *L* (117 mg, 0.625 mmol) in methylene chloride (10 ml). Diffusion between the two phases over a week produced colorless crystals.

### Refinement

H atoms were placed at calculated positions and refined as riding atoms, with H—C = 0.93 Å and with *U*_iso_(H) = 1.2*U*_eq_(C).

### Crystal data

**Table:**

[Ag(NO_3_)(C_18_H_14_N_4_)]	*Z* = 2
*M**_r_* = 456.21	*F*(000) = 456
Triclinic, *P*1	*D*_x_ = 1.767 Mg m^−^^3^
Hall symbol: -P 1	Mo *K*α radiation, λ = 0.71073 Å
*a* = 9.2148 (18) Å	Cell parameters from 3891 reflections
*b* = 9.771 (2) Å	θ = 3.1–27.5°
*c* = 10.800 (2) Å	µ = 1.21 mm^−^^1^
α = 81.51 (3)°	*T* = 293 K
β = 74.27 (3)°	Block, colorless
γ = 66.52 (3)°	0.31 × 0.30 × 0.08 mm
*V* = 857.6 (4) Å^3^	

### Data collection

**Table:**

Rigaku R-AXIS RAPID diffractometer	3891 independent reflections
Radiation source: fine-focus sealed tube	3163 reflections with *I* > 2σ(*I*)
graphite	*R*_int_ = 0.019
Detector resolution: 10 pixels mm^-1^	θ_max_ = 27.5°, θ_min_ = 3.1°
ω scans	*h* = −11→11
Absorption correction: multi-scan (*ABSCOR*; Higashi, 1995)	*k* = −12→12
*T*_min_ = 0.705, *T*_max_ = 0.908	*l* = −13→14
8493 measured reflections	

### Refinement

**Table:**

Refinement on *F*^2^	Primary atom site location: structure-invariant direct methods
Least-squares matrix: full	Secondary atom site location: difference Fourier map
*R*[*F*^2^ > 2σ(*F*^2^)] = 0.032	Hydrogen site location: inferred from neighbouring sites
*wR*(*F*^2^) = 0.094	H-atom parameters constrained
*S* = 1.10	*w* = 1/[σ^2^(*F*_o_^2^) + (0.059*P*)^2^] where *P* = (*F*_o_^2^ + 2*F*_c_^2^)/3
3891 reflections	(Δ/σ)_max_ = 0.002
244 parameters	Δρ_max_ = 0.72 e Å^−^^3^
0 restraints	Δρ_min_ = −0.29 e Å^−^^3^

### Fractional atomic coordinates and isotropic or equivalent isotropic displacement parameters (Å^2^)

**Table:**

	*x*	*y*	*z*	*U*_iso_*/*U*_eq_	
Ag1	1.45799 (2)	0.37548 (2)	0.45603 (2)	0.05309 (11)	
N1	1.2254 (3)	0.3738 (2)	0.5682 (2)	0.0379 (5)	
N2	0.6797 (3)	0.6198 (2)	0.8049 (2)	0.0394 (5)	
N3	0.1476 (3)	1.1322 (2)	1.0027 (2)	0.0451 (5)	
N4	−0.3549 (3)	1.3921 (2)	1.2898 (2)	0.0409 (5)	
N5	1.7214 (3)	0.1260 (3)	0.5841 (3)	0.0519 (6)	
O1	1.8206 (4)	0.0262 (4)	0.6349 (3)	0.0910 (9)	
O2	1.6959 (3)	0.2598 (3)	0.5904 (3)	0.0794 (8)	
O3	1.6440 (4)	0.0949 (3)	0.5224 (3)	0.0686 (7)	
C1	1.2039 (3)	0.2446 (3)	0.5801 (3)	0.0447 (6)	
H1A	1.2915	0.1601	0.5462	0.054*	
C2	1.0576 (4)	0.2313 (3)	0.6405 (3)	0.0530 (7)	
H2A	1.0457	0.1405	0.6437	0.064*	
C3	0.9290 (3)	0.3543 (3)	0.6960 (3)	0.0468 (6)	
H3A	0.8302	0.3470	0.7394	0.056*	
C4	0.9491 (3)	0.4889 (3)	0.6862 (2)	0.0338 (5)	
C5	1.0982 (3)	0.4942 (3)	0.6205 (2)	0.0358 (5)	
H5A	1.1113	0.5850	0.6119	0.043*	
C6	0.8148 (3)	0.6244 (3)	0.7420 (2)	0.0373 (5)	
H6A	0.8299	0.7145	0.7306	0.045*	
C7	0.5498 (3)	0.7521 (3)	0.8539 (2)	0.0356 (5)	
C8	0.4388 (3)	0.7368 (3)	0.9655 (3)	0.0426 (6)	
H8A	0.4528	0.6424	1.0046	0.051*	
C9	0.3077 (3)	0.8597 (3)	1.0195 (3)	0.0464 (7)	
H9A	0.2364	0.8481	1.0960	0.056*	
C10	0.2824 (3)	1.0000 (3)	0.9596 (2)	0.0379 (5)	
C11	0.3917 (4)	1.0142 (3)	0.8472 (3)	0.0492 (7)	
H11A	0.3757	1.1081	0.8065	0.059*	
C12	0.5248 (3)	0.8915 (3)	0.7937 (3)	0.0475 (7)	
H12A	0.5968	0.9031	0.7176	0.057*	
C13	0.0370 (3)	1.1306 (3)	1.0983 (3)	0.0421 (6)	
H13A	0.0434	1.0412	1.1449	0.051*	
C14	−0.1040 (3)	1.2686 (3)	1.1388 (2)	0.0372 (5)	
C15	−0.2228 (3)	1.2683 (3)	1.2502 (3)	0.0405 (6)	
H15A	−0.2108	1.1795	1.2992	0.049*	
C16	−0.3680 (3)	1.5191 (3)	1.2194 (3)	0.0432 (6)	
H16A	−0.4579	1.6051	1.2464	0.052*	
C17	−0.2541 (3)	1.5289 (3)	1.1081 (3)	0.0456 (6)	
H17A	−0.2665	1.6197	1.0622	0.055*	
C18	−0.1215 (3)	1.4008 (3)	1.0666 (3)	0.0408 (6)	
H18A	−0.0447	1.4036	0.9909	0.049*	

### Atomic displacement parameters (Å^2^)

**Table:**

	*U*^11^	*U*^22^	*U*^33^	*U*^12^	*U*^13^	*U*^23^
Ag1	0.03204 (13)	0.06102 (16)	0.05624 (16)	−0.01785 (10)	0.01587 (10)	−0.02131 (11)
N1	0.0264 (10)	0.0419 (10)	0.0382 (11)	−0.0094 (9)	0.0035 (8)	−0.0110 (9)
N2	0.0282 (10)	0.0374 (10)	0.0411 (11)	−0.0048 (8)	0.0019 (9)	−0.0082 (9)
N3	0.0340 (11)	0.0366 (10)	0.0471 (12)	−0.0048 (9)	0.0079 (10)	−0.0068 (10)
N4	0.0270 (10)	0.0442 (11)	0.0430 (12)	−0.0088 (9)	0.0045 (9)	−0.0137 (10)
N5	0.0433 (13)	0.0540 (14)	0.0494 (14)	−0.0170 (12)	0.0032 (11)	−0.0059 (12)
O1	0.0671 (18)	0.092 (2)	0.106 (2)	−0.0279 (16)	−0.0287 (17)	0.0307 (18)
O2	0.0651 (16)	0.0626 (14)	0.111 (2)	−0.0149 (12)	−0.0198 (16)	−0.0331 (14)
O3	0.0859 (19)	0.0556 (12)	0.0735 (16)	−0.0326 (13)	−0.0263 (14)	0.0020 (12)
C1	0.0350 (13)	0.0367 (12)	0.0483 (15)	−0.0050 (11)	0.0031 (11)	−0.0087 (11)
C2	0.0443 (16)	0.0356 (12)	0.067 (2)	−0.0132 (12)	0.0051 (14)	−0.0053 (13)
C3	0.0344 (13)	0.0434 (13)	0.0524 (16)	−0.0143 (11)	0.0057 (12)	−0.0025 (12)
C4	0.0253 (11)	0.0378 (11)	0.0317 (11)	−0.0066 (9)	−0.0010 (9)	−0.0074 (10)
C5	0.0282 (11)	0.0388 (12)	0.0369 (12)	−0.0109 (10)	−0.0007 (10)	−0.0091 (10)
C6	0.0268 (11)	0.0417 (12)	0.0390 (13)	−0.0084 (10)	−0.0023 (10)	−0.0123 (11)
C7	0.0240 (11)	0.0386 (12)	0.0380 (12)	−0.0080 (9)	−0.0003 (10)	−0.0071 (10)
C8	0.0297 (12)	0.0362 (12)	0.0475 (15)	−0.0064 (10)	0.0032 (11)	−0.0002 (11)
C9	0.0316 (13)	0.0423 (13)	0.0453 (15)	−0.0056 (11)	0.0096 (11)	−0.0022 (12)
C10	0.0283 (12)	0.0352 (11)	0.0406 (13)	−0.0074 (10)	0.0033 (10)	−0.0073 (10)
C11	0.0411 (14)	0.0357 (12)	0.0488 (16)	−0.0065 (11)	0.0100 (12)	0.0018 (12)
C12	0.0365 (14)	0.0432 (13)	0.0432 (14)	−0.0090 (11)	0.0132 (11)	−0.0034 (12)
C13	0.0336 (13)	0.0327 (11)	0.0468 (14)	−0.0070 (10)	0.0049 (11)	−0.0053 (11)
C14	0.0269 (11)	0.0363 (11)	0.0413 (13)	−0.0084 (10)	0.0016 (10)	−0.0091 (10)
C15	0.0324 (12)	0.0390 (12)	0.0420 (13)	−0.0117 (10)	0.0026 (11)	−0.0044 (11)
C16	0.0290 (12)	0.0416 (13)	0.0487 (15)	−0.0033 (10)	−0.0023 (11)	−0.0127 (12)
C17	0.0398 (14)	0.0408 (13)	0.0461 (15)	−0.0073 (11)	−0.0069 (12)	−0.0004 (12)
C18	0.0319 (12)	0.0431 (13)	0.0391 (13)	−0.0113 (11)	0.0022 (10)	−0.0052 (11)

### Geometric parameters (Å, °)

**Table:**

Ag1—N1	2.162 (2)	C4—C6	1.474 (3)
Ag1—N4^i^	2.163 (2)	C5—H5A	0.9300
Ag1—O2	2.731 (3)	C6—H6A	0.9300
Ag1—O3	2.704 (3)	C7—C12	1.378 (4)
Ag1—Ag1^ii^	3.1631 (8)	C7—C8	1.386 (3)
N1—C1	1.337 (4)	C8—C9	1.382 (3)
N1—C5	1.347 (3)	C8—H8A	0.9300
N2—C6	1.261 (3)	C9—C10	1.385 (4)
N2—C7	1.417 (3)	C9—H9A	0.9300
N3—C13	1.242 (3)	C10—C11	1.381 (4)
N3—C10	1.419 (3)	C11—C12	1.388 (4)
N4—C16	1.336 (4)	C11—H11A	0.9300
N4—C15	1.351 (3)	C12—H12A	0.9300
N5—O1	1.221 (4)	C13—C14	1.472 (3)
N5—O3	1.240 (4)	C13—H13A	0.9300
N5—O2	1.242 (4)	C14—C18	1.382 (4)
C1—C2	1.378 (4)	C14—C15	1.390 (3)
C1—H1A	0.9300	C15—H15A	0.9300
C2—C3	1.379 (4)	C16—C17	1.385 (4)
C2—H2A	0.9300	C16—H16A	0.9300
C3—C4	1.385 (4)	C17—C18	1.382 (4)
C3—H3A	0.9300	C17—H17A	0.9300
C4—C5	1.382 (3)	C18—H18A	0.9300
			
N1—Ag1—N4^i^	159.44 (9)	C12—C7—C8	119.2 (2)
N1—Ag1—Ag1^ii^	111.41 (6)	C12—C7—N2	123.7 (2)
N4^i^—Ag1—Ag1^ii^	79.97 (7)	C8—C7—N2	117.0 (2)
N1—Ag1—O2	112.93 (10)	C9—C8—C7	121.0 (2)
N1—Ag1—O3	97.63 (10)	C9—C8—H8A	119.5
N4^i^—Ag1—O2	87.07 (9)	C7—C8—H8A	119.5
N4^i^—Ag1—O3	92.98 (9)	C8—C9—C10	120.0 (2)
C1—N1—C5	117.7 (2)	C8—C9—H9A	120.0
C1—N1—Ag1	117.05 (16)	C10—C9—H9A	120.0
C5—N1—Ag1	125.13 (18)	C11—C10—C9	118.7 (2)
C6—N2—C7	120.2 (2)	C11—C10—N3	116.5 (2)
C13—N3—C10	121.8 (2)	C9—C10—N3	124.8 (2)
C16—N4—C15	117.8 (2)	C10—C11—C12	121.4 (3)
C16—N4—Ag1^iii^	122.67 (17)	C10—C11—H11A	119.3
C15—N4—Ag1^iii^	119.45 (18)	C12—C11—H11A	119.3
O1—N5—O3	119.9 (3)	C7—C12—C11	119.6 (2)
O1—N5—O2	122.2 (3)	C7—C12—H12A	120.2
O3—N5—O2	117.9 (3)	C11—C12—H12A	120.2
N1—C1—C2	122.8 (2)	N3—C13—C14	120.8 (2)
N1—C1—H1A	118.6	N3—C13—H13A	119.6
C2—C1—H1A	118.6	C14—C13—H13A	119.6
C1—C2—C3	119.1 (3)	C18—C14—C15	118.7 (2)
C1—C2—H2A	120.5	C18—C14—C13	120.7 (2)
C3—C2—H2A	120.5	C15—C14—C13	120.7 (2)
C2—C3—C4	119.1 (3)	N4—C15—C14	122.5 (2)
C2—C3—H3A	120.5	N4—C15—H15A	118.8
C4—C3—H3A	120.5	C14—C15—H15A	118.8
C5—C4—C3	118.3 (2)	N4—C16—C17	123.2 (2)
C5—C4—C6	120.4 (2)	N4—C16—H16A	118.4
C3—C4—C6	121.3 (2)	C17—C16—H16A	118.4
N1—C5—C4	123.0 (2)	C18—C17—C16	118.5 (3)
N1—C5—H5A	118.5	C18—C17—H17A	120.7
C4—C5—H5A	118.5	C16—C17—H17A	120.7
N2—C6—C4	120.9 (2)	C14—C18—C17	119.3 (2)
N2—C6—H6A	119.6	C14—C18—H18A	120.3
C4—C6—H6A	119.6	C17—C18—H18A	120.3

Symmetry codes: (i) *x*+2, *y*−1, *z*−1; (ii) −*x*+3, −*y*+1, −*z*+1; (iii) *x*−2, *y*+1, *z*+1.

### Hydrogen-bond geometry (Å, °)

**Table:**

*D*—H···*A*	*D*—H	H···*A*	*D*···*A*	*D*—H···*A*
C1—H1A···O3^iv^	0.93	2.50	3.276 (4)	141
C16—H16A···O2^v^	0.93	2.44	3.280 (4)	151

Symmetry codes: (iv) −*x*+3, −*y*, −*z*+1; (v) −*x*+1, −*y*+2, −*z*+2.
